# Splenic Abscess in Qatar: A Single-Center Experience

**DOI:** 10.5339/qmj.2022.16

**Published:** 2022-03-12

**Authors:** Fahmi Yousef Khan, Ahmed Elmudathir, Muhammed Abu Bakir, Bisher Alsawaf

**Affiliations:** Department of Medicine, Hamad General Hospital, Doha, Qatar E-mail: fakhanqal@yahoo.co.uk

**Keywords:** Spleen abscess, splenectomy, percutaneous drainage, antimicrobials

## Abstract

Background & Objectives: Splenic abscess (SA) is a rare clinical entity. There is a lack of information on SA in most Arab and Gulf countries, including Qatar. This study describes the demographics, clinical features, microbiologic etiologies, treatments, and outcomes of patients with SA at the largest tertiary medical center in Qatar over the previous six years.

Methods: This retrospective observational study was conducted at Hamad general hospital. It involved all patients of 18 years old or above who were admitted with the diagnosis of SA for the period between January 1, 2015, and December 31, 2020.

Results: We recruited 25 patients, of which 14 (56%) were males, and 11 (44%) were females. The mean age ( ±  SD) of them was 48.64 ± 19.08 years. The mean illness duration was 22.88 ± 11.88 days. Fever was the most common presenting symptom and was found in 21 (84%) cases, whereas bacteremia was the most predisposing factor found in 15 (60%) patients. The etiology of SA was bacterial in 16 cases (64%), mixed (fungal and bacterial) in one (4%), and tuberculous in one (4%), whereas the etiological agent was unidentified in seven (28%) cases. Intravenous antimicrobial therapy was administered empirically in all patients. However, seven patients (28%) received intravenous antibiotics as the only treatment modality for SA, 15 patients (60%) underwent percutaneous drainage with a pigtail catheter, and two patients underwent splenectomy. The inhospital mortality was three (12%).

Conclusions: This study showed that SA could be caused by various organisms that should be isolated to guide the choice of antimicrobial agents. An abdominal computed tomography is a good diagnostic modality, whereas computed tomography- and ultrasonography-guided percutaneous drainage were efficient therapeutic options that reduce the need for surgery.

## Introduction

A splenic abscess (SA) is an uncommon infection in immunocompetent adults, probably due to the efficient reticuloendothelial system phagocytic activity of the spleen and, consequently, is more likely seen in patients with predisposing factors such as bacterial endocarditis, septicemia, immunologic deficiencies, intravenous drug abuse, splenic trauma, and infarcts.^1–3^ The exact incidence of SA is not well known. However, its prevalence in autopsy studies varies between 0.05% and 0.7%. The frequency of SAs seems to be increasing over recent decades due to the increasing number of severely immunocompromised patients (e.g., AIDS patients and oncologic patients treated with aggressive cancer therapies), an increasing number of patients with traumatic splenic hematoma who are treated conservatively, in addition to the improved diagnostic facilities, in which computed tomography (CT), and ultrasound (US) play a critical role.^1–5^ Untreated or late-treated SA is associated with high mortality, underscoring the need for early detection and prompt therapy to reduce morbidity and mortality from SA. An early diagnosis can easily be made by combining abdominal CT and/or US and clinical features. Treatment of a SA is based on antimicrobial therapy and splenectomy or percutaneous drainage (PD) with good reported results.^1,2,5^

There is a lack of information on SA in most Arab and Gulf countries, including Qatar. The objective of the current study was to describe the demographic characteristics, clinical features, microbiologic etiologies, diagnostic modalities, and treatment outcomes of SA in patients admitted to the largest tertiary medical center in Qatar over the previous six years.

## Methods

### Design, population, and setting

This retrospective observational study was conducted at Hamad general hospital. It involved all patients 18 years old or older who were admitted with the diagnosis of SA for the period between January 1, 2015, and December 31, 2020.

### Data source and data collection

The list of patients was obtained from the medical information system, and the detailed patient data were obtained from their electronic medical records (Cerner). The following data were collected: demographic data, clinical presentation, predisposing factors, etiological agents, diagnostic modalities, lab findings, treatment received, and treatment outcomes.

### Diagnosis of SA

In this study, the SA was identified with imaging modalities, such as an abdominal sonogram, CT, or magnetic resonance image (MRI) of the abdomen, and confirmed by abscess drainage (percutaneous), pathogen isolation from splenic aspirate, or an improvement in the patient's clinical condition after an antimicrobial therapy course.^1,2^

We considered the etiological agent of SA as bacterial, fungal, polymicrobial, tubercular, or unidentified. The etiology was considered as bacterial or fungal if the microorganisms were isolated from blood or drained abscess.^3,4^ Whereas the case was considered splenic tuberculosis if *Mycobacterium tuberculosis* (M. TB) was detected from the drained abscess, or there was an associated TB disease, with an imaging picture consistent with SA that disappeared after a course of antituberculosis therapy.^1,2^ In a patient with an imaging study compatible with a SA that resolved after antimicrobial therapy, but his blood culture or drained abscess culture did not reveal any microorganisms. This case was considered an abscess of unidentified etiology.^1,2,4^

### Ethical approval

The study was approved by the medical research center at Hamad Medical Corporation (Protocol number MRC-01-21-241).

### Statistical analysis

All statistical analyses were performed using SPSS, version 25.0. Categorical and continuous values were expressed as frequency (percentage) and mean ±  SD. Descriptive statistics were used to summarize demographic, epidemiological, laboratory, and other clinical and radiological data of the patients.

## Results

### Demographic and clinical data

During the study period, we recruited 25 patients, of which 14 (56%) were males, and 11 (44%) were females. The mean age of all patients ( ± SD) was 48.64 ± 19.08 years (18–88 years). Ten patients were Qatari, while 15 patients were of other nationalities. The mean illness duration was 22.88 ± 11.88 days (10–60 days). Fever was the most common presenting symptom, noted in 21 (84%) cases, followed by abdominal pain found in 19 (76%) patients. Bacteremia was the most predisposing factor found in 15 (60%) patients, followed by diabetes mellitus 14 (56%). Table 1 describes the main demographic and clinical characteristics of the study patients.

### Etiological factors, investigations, and diagnosis

The etiology of the SA was bacterial in 16 cases (64%), mixed (fungal and bacterial) in one (4%), tuberculous in one (4%), and unidentified in seven (28%) cases. Among patients with bacterial SA, five patients showed Gram-positive infections (one *Enterococcus faecalis*, one *Streptococcus agalactiae*, one *Clostridium perfringens*, one *Streptococcus anginosus*, and one *Staphylococcus aureus*), and 11 had Gram-negative rod infections (five *Escherichia coli*, two *Klebsiella pneumoniae*, one *Parabacteroides distasonis*, one *Fusobacterium nucleatum*, one *Salmonella typhi*, one *Burkholderia pseudomallei*, and one *Pseudomonas aeruginosa*). Table 2 describes the etiological agents of SA in this study. All patients had high CRP 216.59 ± 107.61(95–480)], and most (72%) had leukocytosis (a white blood cell count >10,000/mm^3^). Table 3 summarizes the results of the primary investigations performed in this series.

Chest X-rays showed left-sided abnormalities in 17 (68%) patients with left pleural effusion being the most common finding 15 (88.2%). Of the 25 patients, CT of the abdomen was performed in 22 (88%), abdominal ultrasound in 20 (80%), and MRI in eight (32%). A solitary abscess was observed in 10 (40%) patients, whereas 15 (60%) had multiple SAs. Blood cultures were performed in all patients and SA aspirate cultures in 20 patients to establish the microbiological diagnosis. Table 4 summarizes the diagnostic workup for each patient involved in this study.

### Management and outcomes

Intravenous antimicrobial therapy was administered to all patients. However, seven (28%) patients received intravenous antibiotics as the only treatment modality for SA. Fifteen (60%) patients underwent PD with a pigtail catheter (Table 4), while two patients underwent splenectomy. One patient was initially given antibiotics but later switched to antituberculous therapy based on an AFB smear-positive sputum test. The details of antimicrobials are provided in Table 4. The duration of antibiotic therapy was 4.4 ± 2.2 weeks (1–8 weeks), while antitubercular medications were administered for six months. The overall inhospital mortality was three (12%). The mortality rates in patients treated with antibiotics only, PD, and splenectomy were 0%, 20%, and 0%, respectively (Table 4).

## Discussion

SA is a rare clinical entity whose global incidence is not well known due to the scarcity of studies and most data is derived from case reports. To our knowledge, this is the first study in Qatar and the Gulf countries designed to study this rare clinical condition.

The most common cause of SA is primary hematogenous inoculation from a distant septic source, such as bacterial endocarditis associated with valvular heart disease, intravenous drug use, bacteremia, urinary tract infection, pneumonia, and postoperative or primary intraabdominal infections.^6–8^ Endocarditis is the most classic underlying condition that results in SA, occurring in 10%–20% of cases.^9^ Other underlying conditions include secondary infection of splenic trauma, splenic infarction, and functionally abnormal spleen associated with hemoglobinopathies.^1,6,7,8^ In our study, bacteremia was present in most (60%) patients, whereas endocarditis was confirmed in only 8% of cases.

As noted in this study, fever was the most common presenting symptom, followed by abdominal pain, which is in line with other studies in the literature.^1–9^ However, these symptoms are nonspecific and can be found in other intraabdominal infections. Therefore, early diagnosis of a SA requires a high index of suspicion. Our data showed that 68% of our patients presented with abnormal radiologic signs of left chest, we suggest performing abdominal CT or US as early as possible in patients with unexplained fever, abdominal pain, and abnormal left-sided radiologic findings followed by aspirate and blood cultures.

The causative organisms of SA vary from study to study. A literature review found that SAs can be caused by various organisms that have changed over time.^10^ Brook et al. found that anaerobes were more common as causative organisms than the aerobes, while *E. coli* was the most common isolate among aerobes.^11^ Decades later, Change et al. and Smyrniotis et al.^12,13^ noted that Gram-negative organisms were the most common causative organisms, while Alvi et al.,^14^ found that Gram-positive organisms predominated. Moreover, a report from Spain showed that *Mycobacterium tuberculosis* was the most common pathogen of SA. On the other hand, a recent report,^10^ as well as ours, found that Gram-negative organisms are more common than Gram-positive organisms, while Lee et al. observed that Gram-positive organisms were more commonly involved in SA.^1^

A CT scan is the gold standard for diagnosing SA. The reported sensitivity of CT for this purpose typically approaches 100%.^8,15^ However, given the highly diverse microbiology of SAs, establishing a microbiological diagnosis is essential to manage this clinical entity effectively.^10^ Therefore, SA aspirate and blood cultures should be obtained, and the culture results should be utilized to guide antibiotic choice. We performed blood cultures in all patients, whereas in 20 patients, we applied cultures of aspiration of SA guided by US or CT to establish the microbiological diagnosis in our series (Table 4).

If a SA is suspected, empirical broad-spectrum intravenous antibiotic therapy should be initiated while patients are prepared for surgical drainage or PD. Antibiotic coverage should then be adjusted based on blood culture or abscess results.^16^

There is no gold standard for treating SA as the best therapeutic approach to SA remains controversial.^4,15^ Nevertheless, in recent decades, CT- or US-guided PD has gained acceptance as an effective and less invasive treatment method than surgical intervention^1,4,10,12,15–19^ with a reported success rate ranging from 60% to 100%.^1,2,4,12,15^ It was believed that this procedure would preserve the spleen and thus its immunological function and avoid the risk of overwhelming sepsis after splenectomy.^4,15,16^ In our series, intravenous antimicrobial therapy was administered empirically in all patients, and 60% of them underwent PD with a success rate of 80% (Table 4), which falls within the reported international range of success. Therefore, we agree with Chiang et al.,^15^ who suggested that all cases of SAs should be considered for PD when the risks of surgical drainage are significant or when splenic tissue preservation is essential to avoid the risk of overwhelming post-splenectomy sepsis. Open drainage is sometimes required when PD fails.^8^ In contrast, splenectomy is indicated in cases refractory to PD or response failure to antibiotic therapy alone.^16^ If surgery is performed, a laparoscopic approach is preferred over an open approach.^8^ The role of antibiotic therapy alone to cure SA is controversial as the study results are contradictory. In our study, 28% of our patients received intravenous antibiotics as the only treatment for a SA with a 100% success rate. Noteworthy, the literature review showed that the number of patients who received antibiotics alone to treat SAs ranged from 18% to 68%, with a reported success rate of 80% to 100%.^1,2,4,12,14,20^ However, these results should be interpreted cautiously, and this treatment modality should be tailored and applied to SA patients on an individual basis with close monitoring.

There is no agreement on the duration of antibiotic therapy in patients with SA.^16^ It must be adapted on a case-by-case basis. Patients treated conservatively with antibiotics alone should be monitored closely to ensure complete lesion resolution and adherence to the prolonged course of antibiotics.^20^ If splenectomy is performed and the focus of infection is eradicated, briefer durations may be possible.^16^ In our study, the mean duration of antibiotic therapy was 4.4 ± 2.2 weeks (1–8 weeks). Patients with splenic tuberculosis (TB) should be treated as extrapulmonary TB with the duration of antitubercular therapy from 6–9 months.

Mortality from a SA is variable. If the diagnosis is delayed, SA carries very high mortality that reaches more than 70% and can reach 100% among patients who do not receive antibiotic treatment.^8,21^ However, with appropriate treatment, mortality can be reduced to less than 1%.^8^ In this series, the overall inhospital mortality was three (12%), which is in line with the global range of 0%–27%.^1,4,5,12,14,20^ As noted in our series, the mortality rate in patients treated with antibiotics, PD, and splenectomy only were 0%, 20%, and 0%, respectively. This is in contrast to other reports^1,4^ showing that the mortality rate did not differ between the three groups. Mortality appeared to be more related to the patient's underlying general condition than the procedure performed.

This study is limited by its retrospective design, small sample size, and single-center site. Moreover, due to the study's retrospective nature, most patients were not followed up for 12 months or more after discharge. Nevertheless, we believe that our series will complement the limited data in the literature.

## Conclusion

In conclusion, this study showed that SA was caused by various organisms that should be isolated to guide the choice of antimicrobial agents. In the absence of evidence-based guidelines, the therapeutic approach should be on a case-by-case basis. An abdominal CT is a good diagnostic modality, whereas CT- and US-guided PD were efficient therapeutic options that reduced the need for surgery. Based on our experience, we believe that an interprofessional approach may facilitate prompt diagnosis and efficient treatment.

## Figures and Tables

**Table 1:** Demographic and clinical characteristics of study patients.

Variables	n(%)/mean ± SD (range)

Demographics	

Age	48.64 ± 19.08 (18–88 years)

Sex	

Male	14(56%)

Female	11(44%)

Nationality	

Qatari	10(40%)

Egyptian	3(12%)

Jordanian	2(8%)

Sudanese	2(8%)

Nepalese	2(8%)

Filipino	2(8%)

Syrian	1(4%)

Saudi	1(4%)

Bahraini	1(4%)

Indian	1(4%)

Clinical data	

Duration of symptoms	22.88 ± 11.88 (10–60 days)

Fever	21(84%)

Abdominal pain	19(76%)

Nausea/vomiting	8(32%)

Chills	8(32%)

Anorexia	7(28%)

Weight loss	6(24%)

Cough	4(16%)

Chest pain	4(16%)

Diarrhea	3(12%)

Splenomegaly	13(52%)

Hepatomegaly	9(36%)

Predisposing factors	

Bacteremia	15(60%)

Diabetes mellitus	14(56%)

Heart failure	5(20%)

Active cancer	4(16%)

Urinary tract infection	4(16%)

End stage renal disease	4(16%)

Chronic obstructive pulmonary diseases	3(12%)

Steroid therapy	3(12%)

Infective endocarditis	2(8%)

Splenic trauma	2(8%)

Others*	10(40%)


*Others: acute pancreatitis, chronic liver disease, systemic lupus erythematous, fungemia, cerebral palsy, hypertension, postpartum hemorrhage, pneumonia, renal transplantation, post-gastric sleeve splenic infarction

**Table 2:** Etiological agents of splenic abscess

Etiological agents	n(%)

Unidentified	7(28%)

*E coli*	5(20%)

*Klebsiella pneumonia*	2(8%)

*Parabacteroides distasonis*	1(4%)

*Enterococcus faecalis*	1(4%)

*Streptococcus agalactiae*	1(4%)

*Clostridium perfringens*	1(4%)

*Fusobacterium nucleatum*	1(4%)

*Salmonella typhi*	1(4%)

*Mycobacterium tuberculosis*	1(4%)

*Streptococcus anginosus*	1(4%)

*Staphylococcus aureus*	1(4%)

*Burkholderia pseudomallei*	1(4%)

Candida orthopsilosis + *Pseudomonas aeruginosa*	1(4%)


**Table 3:** Investigations

Investigations	Mean ± SD (range)

WBC	14480 ± 9700 (3400–45000/mm^3^)

Leukocytosis	18(72%)

Hemoglobin	10.45 ± 1.65(8-14 g/dL)

Anemia	20(80%)

CRP	216.59 ± 107.61(95–480)

Procalcitonin	5.11 ± 8.62(0.04–43.60)

Lactic acid	2.28 ± 0.85(0.9–4.0)


CRP: C-reactive protein; WBC: white blood cells; SD: standard deviation

**Table 4:** Demographics, clinical data, and treatment outcomes of 25 patients with splenic abscess.

Case no.	Age (years)	Sex	Duration of symptoms (days)	Predisposing factors	Number of abscesses	Microbiological etiology and diagnosis	Treatment	Duration of antimicrobials (weeks)	Radiologic resolution	Outcome

1	68	M	14	Heart failure, DM, active cancer	Multiple	Unidentified	Ab	4	Yes	Recovered

2	71	F	30	DM, ESRD, bacteremia	Single	*E. coli* (abscess and blood)	Ab+PD	6	Yes	Recovered

3	75	M	25	Heart failure, DM, ESRD, COPD, active cancer	Single	*E. coli* (abscess)	Ab+PD	1	Not applicable (Died)	Fatal

4	76	F	28	DM, ESRD, bacteremia	Multiple	*Parabacteroides distasonis* (blood and abscess)	AB+PD	1	Not applicable (Died)	Fatal

5	88	M	14	COPD, Heart failure, DM, HTN	Single	Unidentified	Ab+ ST	2	Not applicable (Splenectomy)	Recovered

6	46	F	20	DM	Multiple	Unidentified	Ab	7	Yes	Recovered

7	28	M	36	Cerebral palsy, acute pancreatitis	Single	Unidentified	Ab+PD	8	Yes	Recovered

8	58	M	18	Infective endocarditis, bacteremia, spleen trauma	Multiple	*Enterococcus faecalis* (blood and abscess)	Ab+PD	8	Yes	Recovered

9	46	M	16	DM, infective endocarditis, bacteremia, pneumonia	Multiple	*Streptococcus agalactiae* (blood)	Ab	7	Yes	Recovered

10	49	M	14	DM, active cancer, bacteremia	Multiple	*Clostridium perfringens* (blood)	Ab+PD	5	Yes	Recovered

11	43	F	30	Bacteremia	Single	*Fusobacterium nucleatum* (blood)	Ab+PD	4	Yes	Recovered

12	32	F	50	Bacteremia, UTI, post-partum hemorrhage	Multiple	*E. coli* (blood and urine)	Ab	6	Yes	Recovered

13	31	F	10	Bacteremia, acute pyelonephritis	Single	*E. coli* (blood and urine)	Ab	2	Yes	Recovered

14	27	M	20	Spleen trauma, bacteremia	Single	*Klebsiella pneumoniae* (blood and abscess)	Ab+PD	3	Yes	Recovered

15	30	F	13	SLE, on steroid, UTI	Multiple	Unidentified	Ab	6	Yes	Recovered

16	25	M	16	Typhoid bacteremia	Multiple	*Salmonella typhi* (blood and abscess)	Ab+PD	4	Yes	Recovered

17	23	M	28	None	Multiple	*M. tuberculosis* (sputum)	Anti TB	30	Yes	Recovered

18	44	F	14	DM, bacteremia, UTI	Single	*Klebsiella pneumonia* (blood and abscess)	Ab+PD	4	Yes	Recovered

19	65	M	18	DM, renal transplant on steroid, Heart failure, bacteremia	Single	*E. coli* (blood and abscess)	Ab+PD	6	Yes	Recovered

20	51	M	30	DM	Multiple	*Streptococcus anginosus* (abscess)	Ab+ST	2	Not applicable	Recovered

21	68	M	20	DM, active cancer, bacteremia	Single	*Staphylococcus aureus* (blood)	Ab+PD	6	Yes	Recovered

22	54	M	18	Bacteremia, DM	Multiple	*Burkholderia pseudomallei* (blood)	Ab	6	Yes	Recovered

23	45	F	15	DM, heart failure,	Multiple	Unidentified	Ab+PD	4	Yes	Recovered

24	18	F	60	Post-gastric sleeve splenic infarction	Multiple	Unidentified	Ab+PD	6	Yes	Recovered

25	55	F	15	DM, COPD on steroid, bacteremia, fungemia	Multiple	*Candida orthopsilosis, Pseudomonas aeruginosa* (blood)	Ab+AF+ PD	1	Not applicable (Died)	Fatal


M: male; F: female; DM: diabetes mellitus; COPD: chronic obstructive pulmonary disease; HTN: hypertension; SLE: systemic lupus erythematous; UTI: urinary tract infection; Ab: antibiotics; PD: percutaneous drainage; ST: splenectomy; AF: antifungal; TB: tuberculosis
